# Supplementary material for: Are carbon emissions trading and green financial instruments synergistic? -Comprehensive quantitative research based on content analysis
Source: PLoS One. 2024 Mar 7;19(3):e0298601. doi: 10.1371/journal.pone.0298601 (PMC10919601; doi:10.1371/journal.pone.0298601)
Supplement: S1 Data — (ZIP) [file pone.0298601.s001.zip › Data/地级市Z_F工作报告.zip!/╔╜╬≈/┴┘╖┌╩╨/└┤╫╘╣1⁄2╓┌║┼í╛┬φ┐╦╩2╛▌═°í┐_2018.docx]

各位代表：

　　现在，我代表市人民政府向大会报告工作，请予审议，并请市政协委员和其他列席人员提出意见。

　　一、2017年工作回顾

　　过去的一年，是我市发展进程中极不平凡的一年。面对经济下行和环保倒逼的双重压力，在省委、省政府和市委的坚强领导下，在市人大、市政协的监督支持下，市政府坚持以习近平新时代中国特色社会主义思想为指引，坚决维护习近平总书记核心地位，坚决维护党中央权威和集中统一领导，坚决贯彻党的十九大精神和习总书记视察山西重要讲话精神，不断增强“四个意识”，坚定“四个自信”，深入落实新发展理念，紧紧围绕省委“一个指引、两手硬”的思路和要求，聚焦“345”发展战略和“12345”工作思路，改革创新，奋发作为，全市经济社会发展取得了新成绩。

　　——坚持稳中求进，经济形势总体向好。全市经济增速从一季度走出困难时期，到上半年稳步回升，再到前三季度步入合理区间，全年实现稳步增长。地区生产总值完成1320.1亿元，比上年增长5.5%，为四年来最好水平;规模以上工业增加值完成413亿元，比上年增长1%;固定资产投资完成578.9亿元，比上年增长6.9%，增速位居全省第二;社会消费品零售总额完成653.1亿元，比上年增长7.2%，增速位居全省第三;一般公共预算收入完成97.1亿元，比上年增长12.9%;城镇常住居民人均可支配收入完成28873元，比上年增长6.6%;农村常住居民人均可支配收入完成10742元，比上年增长7.4%，各项指标呈现快速增长的良好态势，经济发展后劲不断增强，全面建设小康社会迈出新步伐。

　　——坚持转型升级，动能转换明显加快。工业产业转型提速。围绕构建“4+6+N”现代工业体系，重点实施了61个工业转型升级项目，通才工贸高温超高压煤气发电、汤荣机械制造双金属复合一体制动毂等28个项目完工，华为(临汾)云计算大数据产业园完成规划、设计、选址等前期工作，临汾“互联网+”智慧产业园项目全面启动实施。全市战略性新兴产业、装备制造业、高技术产业分别增长55.1%、63.9%、46.3%，成为工业经济增长的主要拉动力。现代农业加快发展。粮食产量达52.1亿斤，又是一个丰收年。新发展和改造设施蔬菜2.1万亩、果园15.8万亩、中药材22.1万亩。新发展养猪项目17个，新建晋南牛等养牛基地15个。肉、蛋、奶类产量分别达33.4万吨、17.5万吨、6.2万吨。全年培育壮大农产品加工龙头企业29家，农产品加工销售收入完成100.2亿元，增幅达26.6%。蒲县成功入选全国首批“国家级农业可持续发展试验示范区暨农业绿色发展试点先行区”。强力打造以尧都、侯马、霍州为中心的现代都市休闲农业圈，农村新业态、新模式蓬勃发展。现代服务业发展迅猛。商贸、电商、文旅等产业加快发展。设立1000万元加工贸易产业转移引导资金，承接加工贸易转移取得新突破。汾西、隰县被商务部确定为“电子商务进农村综合示范县”。全市接待国内外游客人数达5255.4万人次，旅游总收入达到484.5亿元，同比增长30%。三次产业比重优化为7.2∶46.3∶46.5，第三产业对经济增长的贡献率达到70.2%。

　　——坚持城乡融合，基础设施日臻完善。城市品位不断提升。全面启动《临汾市市域城镇体系规划(2017-2035)》《临汾市城市总体规划(2017-2035)》编制工作，持续加大中心城市建设，实施了40项重点城建工程，特别是滨东、滨西两座立交桥竣工通车，有力提升了市区通行能力，城市立体化交通建设迈出重要步伐。城乡面貌明显改善。扎实开展城乡环境集中整治行动，受到省政府通报表扬。乡村清洁工程省级达标村实现全覆盖，襄汾县等5个农村垃圾治理试点县建设扎实推进。以“四美三宜”为标准，积极参与“三级联创”美丽宜居示范村创建，其中创建省级示范村13个、市级示范村25个。交通建设全面提速。长临高速路基、霍永高速永和至永和关段路基全线贯通。新改建农村公路2050公里，尧都区荣获全国“四好农村路”示范县(区)。沿黄一号旅游公路永和乾坤湾试验段在全省率先开工。新增新能源公交车110辆，临汾“掌上公交”正式运行，成功入选国家“公交都市”示范城市。方略保税物流被确定为全国多式联运示范工程企业，兴荣物流被确定为全国甩挂运输试点企业。民航机场新开通北京、上海、广州等7条航线，通达10个城市，旅客吞吐量突破30万人次。水利工作成效显著。“河长制”体系全面建立，“一河一策”启动实施。引沁入汾、引沁入浮、禹门口东扩、中部引黄、县域小水网工程建设进展顺利，水利支撑作用充分发挥。完成农田实灌面积255.4万亩，新增水土流失治理面积67.5万亩，新增高效节水灌溉面积9.8万亩。23.6万农村人口稳定解决饮水安全问题。

　　——坚持绿色发展，生态治理持续深入。节能减排财政政策综合示范工作深入推进，能耗降幅位居全省前列。围绕解除环保限批、配合中央环保督察等重点工作，以前所未有的决心、力度和措施，大打了一场生态环境治理攻坚战。对中央、省环保督察和自查自纠发现的1560个问题，明确主体、标准、时限，狠抓整改，取得明显成效。强力推进实施“八大工程”，累计完成投资118亿元，为2016年的4倍。完成工业企业深度治理361家，取缔、关停和整治“散乱污”企业865家，取缔和整治燃煤锅炉5035台，完成“煤改气”“煤改电”、集中供热清洁取暖改造19.8万户。秋冬季大气污染防治成效显著，空气质量综合指数、二氧化硫浓度、细颗粒物浓度、可吸入颗粒物浓度、重污染天气数等五项指标降幅均居全省第一。在全省首家成立环境安全保卫支队和环境监察大队，严厉查处各类环境违法行为，在全市形成了铁腕治污的高压态势。出台了《地表水环境综合整治实施方案(2017-2020)》，实施了汾河、浍河、涝洰河等重点河段生态环境综合整治，全市地表水Ⅲ类和劣Ⅴ类水体比例均达到省考核要求，沁河水质达地表水Ⅱ类标准。启动《临汾市土壤污染治理与修复规划》编制工作，土壤污染防治试点工作扎实开展。推进生态造林工程，完成营造林61.3万亩。通过不懈努力，解除了环保限批，生态立市理念进一步确立，环保倒逼转型初见成效，环境质量持续改善。侯卫东官场笔记

　　——坚持改革开放，发展活力有效释放。供给侧结构性改革有力推进。扎实推进“三去一降一补”，压减钢铁产能170万吨、煤炭产能420万吨，去产能工作走在了全省前列;工业产成品存货比上年下降11.4%，商品房去库存消化周期缩短3.4个月;工业企业资产负债率降低2.9%;工业企业每百元主营业务收入成本降低6.7元;清理规范政府性基金15项，削减涉企行政事业性收费53项。综改示范区建设加快推进。贯彻落实国发42号和晋发49号文件精神，制定《临汾市进一步深化改革促进资源型经济转型发展行动计划》，临汾侯马综合保税区已上报国务院待批，临汾、侯马经济开发区扩区和洪洞经济开发区设立已经省政府批准，襄汾、霍州、安泽经济开发区设立已通过省级评审，“多规合一”、机构改革、“三化三制”等工作扎实推进。农业农村改革步入“全省第一方阵”，国有林场、集体林权制度配套、市场监管体制、旅游体制、国企国资等重点领域改革均取得积极进展。对外开放步伐加快。积极融入国家发展战略，我市8个县(市、区)被列入关中平原城市群。围绕打造内陆地区对外开放新高地，加强与京津冀、环渤海、黄河金三角等区域交流合作。不断加强对外贸易，累计开行4列中欧班列，全市海关进出口总额达16.7亿元，比上年增长34.5%，增速位居全省第一。招商引资成效喜人。通过打造“六最”营商环境，临汾成为投资热土。举办各类专题招商推介会15场，签约项目332个，总投资额2213.7亿元，到位资金404.5亿元，大宁县医用手套、蒲县生猪养殖和晋南牛产业化、侯马经济开发区金葵花大健康产业等项目，实现了“当年签约、当年落地、当年开工”。创新驱动不断加力。引深产学研合作，我市先后与山西师范大学、山西中医药大学等院校建立了战略合作关系，华翔集团与太原理工大学、华中科技大学合作建立了材料成型技术与装备研究院、3D打印与绿色铸造实验室。大力推进“双创”工作，培育省级及以上重点实验室和工程技术研究中心4家、科技企业孵化器1家、众创空间8家、高新技术企业42家。“隰县电商孵化众创空间”等3家被认定为国家级“星创天地”。新培育小微企业3160户、“专精特新”企业40户、“管理标杆”企业21户。首次设立“市长创新奖”，16家科技创新优秀企业获奖。大力弘扬劳模精神，积极开展“平阳工匠”评选活动。华翔集团“人人创新、全员创客”模式受到李克强总理的充分肯定并在全国推广。

　　——坚持共建共享，民生福祉不断增强。始终把人民群众对美好生活的向往作为工作的出发点和落脚点，坚持民生优先的工作导向，持续加大民生投入，全年民生支出累计执行268.7亿元，同比增长3.3%，占一般公共预算支出的比重达80.4%。脱贫攻坚再战再胜。扎实推进省市重点扶贫工程，出台了《临汾市聚焦深度贫困集中力量攻坚三年行动计划》，累计投入各类专项扶贫资金12.8亿元，完成225个贫困村退出、8.5万贫困人口脱贫，吉县、乡宁、安泽3个贫困县即将“摘帽”。教育事业优先发展。侯马、洪洞、汾西、曲沃、襄汾5个县市顺利通过国家义务教育均衡验收。“三优”评选活动扎实开展，二本B类以上达线率45.1%，教育教学质量进一步提升。山西师大新校区开工建设，师大临汾学院新校区工程稳步推进。卫生健康事业加快发展。深入推进医药卫生体制改革，17个县(市、区)全部成立医疗集团并挂牌运行，在全省率先实现全覆盖。城市公立医院全部取消药品加成，在北京等21个省(市、自治区)实现医保异地直接结算。全面落实县域内先诊疗后付费一站式服务。重点人群家庭医生签约率达90.6%。“创卫”工作扎实有序推进。文旅事业繁荣发展。成功申办2018年全省旅发大会，陶寺遗址入选国家考古遗址公园立项名单，《唱响新时代》走进山西临汾和元宵特辑在央视播出，临汾影响力进一步提升。成功申报省级非物质文化遗产项目22项，《赵城金藏》复制工程首批经卷顺利回归。“免费送戏下乡”近2000场。竞技体育在全运会上实现了金牌“零”的突破。市图书馆、博物馆基本建成，我市与国家图书馆签署加强临汾市文化建设战略合作框架协议。就业保障扩面提质。全市城镇新增就业53148人，转移农村劳动力57489人，建档立卡农村贫困劳动力免费职业培训8685人，五大保险参保人数和基金征缴全部超额完成省定目标任务。社会大局安全稳定。大力开展安全生产“反三违”专项行动、安全生产大检查和百日攻坚行动，工矿企业连续8年未发生重大事故，安全生产形势持续稳定好转。扎实推进“平安临汾”“法治临汾”建设，我市被确定为全省唯一的法治创建示范市，安泽县荣获全国综治最高奖“长安杯”。

　　——坚持依法履职，自身建设不断提升。政治建设全面加强。深入开展“讲政治、知敬畏、守规矩”专项治理活动和“两学一做”学习教育常态化制度化、“维护核心见诸行动”主题教育等专项活动，党员干部“四个意识”不断增强，执行中央、省委和市委决策部署更加坚决有力，营造了树正气、讲团结、聚合力、促转型的浓厚氛围。依法行政有序推进。扎实推进法治政府建设，严格执行人大及其常委会的决议决定，主动接受人大、政协监督，全年共办理省人大代表建议7件、市人大代表议案建议144件，市政协委员提案234件、意见7件，办结率达100%;围绕环境治理工作主动接受市人大代表的监督和质询;完成《临汾市禁止燃放烟花爆竹规定》《临汾市燃煤污染防治规定》《临汾市旅游资源开发利用保护办法》《临汾市非物质文化遗产保护管理办法》等4部地方性法规的起草、审核、议案报送，经市人大常委会审议通过，全部颁布实施。调查研究深入开展。先后组织开展了市政府领导、市直部门主要负责同志、县(市、区)长3个领题调研活动，积极参与全省“万名干部大调研”活动，形成调研报告455篇，进一步提升了各级领导干部把握地情、推动工作的能力。服务水平不断提高。深入开展“万名干部入企服务”和“万名干部乡镇挂职”工作，服务企业和基层能力进一步提高。加强市政务服务中心建设，制定《政务服务标准体系》，精简、下放审批项目23项，进驻事项平均办理时限从20个工作日压缩至8.5个工作日，压缩率达42.5%，顺利通过国家级政务服务标准化试点中期评估，12345市长热线荣获全国市民热线“先锋奖”。大力推进移动OA办公，推行“13710”工作制度，开通了临汾政务微博，政府信息化建设水平不断提升。管党责任全面压实。市政府党组紧紧抓住党风廉政建设主体责任这个“牛鼻子”，认真落实中央八项规定，持之以恒纠正“四风”，严格执行国务院“约法三章”，围绕加强党的建设和落实“一岗双责”，制定出台相关方案、制度、规定14个，开展集体约谈、提醒谈话和诫勉谈话23次514人次，切实把从严管党治党责任传导压实到岗、到人、到位、到底。

　　与此同时，国防动员、双拥共建和优抚安置工作扎实推进。民族宗教、外事侨务、老龄、残疾人、红十字会、妇女儿童等事业取得新进步，人防、统计、食药监、工商、质监、审计、地方志、水文、气象、地震、档案、住房公积金等工作取得新进展，对口援疆工作取得新成绩。

　　各位代表，一年砥砺奋进，一年硕果累累。这是坚定不移贯彻落实习近平新时代中国特色社会主义思想的结果，是省委、省政府和市委正确领导的结果，是市人大、市政协监督支持的结果，是全市上下奋力推动转型、同心共促发展的结果。在此，我代表市政府，向全市人民，向人大代表、政协委员，向各民主党派、工商联、无党派人士和人民团体，向驻临部队、武警官兵和所有关心支持临汾发展的各界朋友，表示崇高的敬意和衷心的感谢!

　　各位代表，安不忘危，稳不忘忧。在肯定成绩的同时，我们也清醒地认识到，我市发展不平衡不充分问题仍然突出，与人民群众日益增长的美好生活需要仍有很大差距。长期积累的“一煤独大”的结构性矛盾仍十分突出，资源消耗大，发展质量和效益不高，新动能支撑不足;对外开放总体水平不高，发展环境需进一步改善和优化;科技和人才要素支撑不够，整体创新能力不强;城乡区域发展不平衡，脱贫攻坚任务仍十分艰巨;生态环境还比较脆弱，大气、水、土壤环境问题依然严峻，生态保护和修复任务繁重，一些环境污染的深层次矛盾尚未有效解决;民生社会事业欠账较多，补短板任务艰巨;安全生产基础依然薄弱，基层社会治理能力有待提高;政府职能转变还不到位，少数干部法治意识、责任意识、服务意识还不够强等等。对此，我们一定要本着对人民高度负责的精神，采取有力举措，切实加以解决。

　　二、2018年目标任务和重点工作

　　今年是全面贯彻党的十九大精神的开局之年，是改革开放40周年，是决胜全面建成小康社会、实施“十三五”规划承上启下的关键一年，也是我市加快转型升级、推动高质量发展的攻坚之年。

　　今年政府工作的总体要求是：全面贯彻党的十九大、十九届二中、三中全会和全国“两会”精神，以习近平新时代中国特色社会主义思想为指引，在市委的坚强领导下，坚持稳中求进工作总基调，坚持新发展理念，坚持以人民为中心的发展思想，紧扣社会主要矛盾变化，按照高质量发展的要求，统筹推进“五位一体”总体布局和协调推进“四个全面”战略布局，紧紧围绕建设“示范区”“排头兵”“新高地”的目标要求，深入实施“345”发展战略和“12345”工作思路，坚持把深化供给侧结构性改革与深化转型综改试验区建设结合起来，作为经济工作的主线，大力推进改革开放，推动质量变革、效率变革、动力变革，打好防范化解重大风险、精准脱贫、污染防治“三大攻坚战”，全力做好稳增长、促改革、调结构、惠民生、防风险等各项工作，加快经济强市、生态强市、文化强市建设，促进全市经济社会持续健康发展。

　　主要预期指标是：地区生产总值增长6.5%;规模以上工业增加值增长6%左右;全社会固定资产投资增长9%左右;社会消费品零售总额增长7%左右;一般公共预算收入增长7%;城镇常住居民人均可支配收入增长6.5%;农村常住居民人均可支配收入增长6.5%以上。

　　约束性指标是：万元地区生产总值能耗下降3.2%;万元地区生产总值二氧化碳排放量下降3.9%;万元地区生产总值用水量下降指标、环境质量指标和主要污染物总量减排指标完成省定年度目标任务。

　　围绕以上目标，今年重点抓好以下十项工作：

　　(一)加快推进产业转型

　　按照习总书记“推动经济高质量发展，重点是推动产业结构转型升级”的要求，加快构建现代工业产业体系，支持推动民营经济发展，大力实施创新驱动战略，争当全省转型发展排头兵。

　　加快构建现代工业产业体系。着力推进“4+6+N”工业体系建设，打造以开发区和园区为载体的新兴产业集群，强力推进高质量发展，用三到五年时间，将我市建成国家新型综合能源基地、中西部精密铸造生产基地、全省产业转型升级先行区。改造提升传统产业。加快推进传统产业提质增效，在优化存量上下功夫。煤炭行业，坚持“淘汰落后、发展先进、优化结构”的原则，用两年时间完成年产能60万吨以下矿井的减量重组，今年煤炭先进产能占比达到50%以上。钢铁行业，以减量置换升级为主，推动存量产能向优势企业集中，重点打造千万吨级的晋南钢铁集团，加快建设曲沃、襄汾两个钢铁基地，走集约型、集团化发展道路。焦化行业，编制完善《临汾市焦化行业中长期发展规划(2018-2025)》，稳步推进节能环保大焦炉建设，重点打造洪洞、襄汾、安泽三大焦化园区，延伸化产深加工产业链。电力行业，积极发展高效、清洁、绿色煤电，淘汰燃煤机组容量3.6万千瓦，推进隰县盾安98兆瓦风力发电、新疆金风科技风电设备制造及风场运营维护等项目。培育壮大新兴产业。加快推进新兴产业集群式发展，在做大增量上下功夫。现代煤化工产业，围绕“醇、烯、气、苯、油、氨”等产品，发展精细高端化工，重点实施黑猫集团1000万吨精细煤化工等项目。现代装备制造产业，充分发挥我市资源优势，打造临开(华翔)、襄汾、翼城、侯马、尧都五大特色铸造工业园区，培育汽车零部件和车桥总成等优势产品，重点推进华翔增材制造、汤荣环保清洁原材料生产基地和双金属复合一体式制动毂技术改造等项目。新能源产业，加快编制《临汾市综合能源基地建设规划》。加快推进临汾、霍州经济开发区新能源产业园区建设，推进霍州亿能电动汽车等项目，推动新能源汽车产业提质升级、链条延伸。新材料产业，推动新型建材、高端金属结构材料、特种金属功能材料等产业向规模化、高端化、效益化发展，重点推动翼城甲阳国际中空纤维膜、侯马北铜铜精矿综合回收等项目尽快落地建设。新医药产业，以道地中成药为特色、以专利特效药为拳头、以新药研发为重点，扶持旺龙、云鹏等一批医药龙头企业做大做强，推进金葵花医疗器械生产二期等项目。节能环保产业，以可再生能源利用、节能技术装备、半导体照明为重点，加快推进华基建材建筑垃圾及工业尾矿资源化利用等项目。大力发展前沿产业。加快推进“互联网+先进制造+现代服务业”，发展大数据、云计算、生物科技、遥感遥控、空间科技、通用航空等新产业、新业态，大力推进人工智能技术，重点实施华为(临汾)云计算大数据产业园、晋南北斗导航等项目。

　　大力发展民营经济。认真贯彻实施国家新修订的《中小企业促进法》，全面落实好我市《关于进一步促进中小微企业创业创新转型发展的实施意见》，坚决破除各种隐性壁垒，建立健全服务协同机制，尊重、支持和爱护企业家，激发和保护企业家精神，持续构建亲清新型政商关系，全力扶持民营企业发展。按照“个转企、小升规、规改股、股上市”的路径，新培育规上企业20户以上，10家企业在山西股权交易中心挂牌交易，10家中小企业进入股改程序，争取1-3家企业在新三板上市，稳步推进华翔集团整体上市。新培育小微企业3000户以上，认定30个“专精特新”、20个“管理标杆”中小企业，全面提升中小微企业核心竞争力。

　　实施创新驱动战略。继续实施“市长创新奖”，大力推进人才强市，吸引更多创新型、高科技人才落户临汾。培育100名科技型领军人才、500名创新型技术人才、2000名技能型实用人才。大力弘扬工匠精神，培育更多“平阳工匠”。推广华翔集团“双创”经验，激发全社会“双创”热情和活力。推动产学研深度融合，加快推进与中科院北京分院、太原理工大学等院校合作项目落地，促进科技成果向现实生产力转化，年内高新技术企业达44家，省级以上技术中心企业达27家，培育省级及以上重点实验室和工程技术研究中心5家、科技企业孵化器2家、众创空间9家。加强科技管理队伍建设，提升工作水平。

　　各位代表，雄关漫道真如铁，而今迈步从头越。推进转型发展需要持之以恒的耐力，需要善谋善成的方法。我们要树立“功成不必在我”的思想境界，坚持统筹谋划，系统推进，立足当下，着眼长远，坚持不懈，力争交出让人民满意的转型发展新时代答卷!

　　(二)全面推进改革开放

　　“改革开放是决定当代中国命运的关键一招，实践发展永无止境，改革开放也永无止境”。要全面落实好、完成好党和国家机构改革的部署要求，统筹推进以供给侧结构性改革为重点的深层次、多领域改革，进一步扩大对外开放，努力打造内陆地区对外开放新高地。

　　深化供给侧结构性改革。坚持把经济发展着力点放在实体经济上，扎实推进“三去一降一补”。坚持用市场化法治化手段，严格执行环保、质量、安全等标准，化解过剩产能，淘汰落后产能。大力开展银企对接活动，充分发挥“临汾市企业资金链应急周转保障资金”的助企作用，稳定企业信贷规模，降低企业融资成本。健全完善企业税费清单制度，落实好减轻实体经济负担的政策措施，切实降低企业运营成本。坚持问题导向，围绕全市经济社会发展薄弱环节精准施策，补齐短板，进一步提高供给的精准性、有效性。

　　深化开发区体制机制改革。积极推进“多规合一”，完善市域城镇体系规划调整，加快编制各开发区总体规划、控制性详细规划。突出抓好设区升级工作，整合吉县、乡宁、大宁、永和、隰县、蒲县等沿黄6县的旅游资源，设立沿黄生态文化旅游带;加快临汾侯马综合保税区设立及建设，推进临汾经济开发区升级，争取早日获批国家级经济开发区;加快霍州、襄汾、安泽、翼城、汾西5个经济开发区和临汾清洁能源高效利用示范区申报省级经济开发区工作，力争霍州、襄汾、安泽获批。高标准推进新设立开发区的起步区建设，完善基础设施，做好“九通一平”。深入推进“三化三制”改革，临汾、侯马经济开发区“三制”改革全部到位，“三化”改革持续推进。

　　深化农业农村改革。完成农村土地确权改革扫尾工作，建设农村承包地确权数据库信息平台。推进农村承包地“三权分置”改革，加强县、乡农村产权流转交易市场建设，落实二轮土地承包到期后再延长30年等政策措施，制定我市“以奖代补”扶持土地流转政策。推进农村集体产权制度改革，省、市试点县(村)年内完成改革任务。推广洪洞县农业水价综合改革试点经验，促进节水型社会建设。

　　深化国企国资改革。实施国有企业改革振兴计划，完善国企国资改革政策体系。以交通、煤炭为试点，组建市属国有资本投资运营平台，提升国有资本管理能力，有条件的县(市、区)组建投资运营公司。全面推进企业“处僵治困”，依法处置债权债务，加大对低效无效资产清理整顿力度。持续推进剥离企业办社会工作，年底前基本完成国有企业职工家属区“三供一业”、市政、社区、消防、教育、医疗机构的分离移交工作。

　　深化市场、商事、财税等领域改革。市场监管方面，加快推进职能、机构、队伍、平台等整合，努力实现行政审批、执法办案、市场监管、公众诉求处理、检验检测、信息保障“六个一体化”。商事制度方面，全面梳理涉企证照事项，不断扩大“多证合一”覆盖面。大力推行市场主体登记全程电子化和电子营业执照，推进企业名称登记管理改革，积极落实住所申报承诺制。财税体制方面，加大预算统筹力度，扩大预算公开范围。启动中期财政规划，全面实施绩效管理，使财政资金花得其所、用得安全。质量标准方面，大力实施质量强市战略，依托山焦、华翔、戎子酒庄等企业，在制造业、工业制成品等领域提升标准。加强质量监管，实施质量奖励制度，打造一批工业类、服务类和工程类品牌。统筹推进金融体制、国有林场、集体林权制度配套、医药卫生体制、电力体制等改革。

　　扩大对外开放。坚持以全面提升对外开放水平为目标，着力打造一个平台，建设两个港口，用好四条通道，创新五个招商，全方位、宽领域、多层次推进对外开放。打造一个平台。紧紧抓住我市列入关中平原城市群的良好机遇，依托方略保税物流中心建设综合保税区，全力打造我市对外开放新平台。建设两个港口。加快推进侯马无水港和临汾空港建设，更好地发挥港口对物流、资金流、信息流等资源要素集聚辐射作用，提升对外贸易水平。用好四条通道。坚持用好中欧班列、五定班列、中南铁路、国际航空口岸四条通道，加快完善进出口网络体系，深度融入“一带一路”和京津冀、环渤海、黄河金三角等区域，加强与德国、波兰等欧洲国家的商贸交流，推动我市与其他国家和地区更高水平的互联互通。创新五个招商。坚持以商招商，依靠本地企业吸引外地企业投资，内外合作实现共赢;坚持以链招商，通过延伸上下游产业链条引进项目，形成产业集群;坚持以区招商，把开发区、产业园区作为我市招商引资的大平台，充分利用独特的政策、环境、资源优势引进项目;坚持以情招商，带着感情抓招商，以跟踪服务、贴心服务、精细服务换“商机”;坚持以县招商，以县(市、区)为主体进行招商引资，变集中行动为靶向招商，提高招商引资的针对性和有效性。

　　各位代表，改革开放，任务艰巨。但惟其艰难，方显勇毅，惟其磨砺，始得玉成。我们要勇于啃硬骨头，敢于战胜一切困难和风险，以更实的举措、更大的力度、更加包容的姿态，推动改革开放迈出新步伐，为高质量发展积蓄能量，开拓空间!

　　(三)持续推进项目建设

　　全面贯彻落实全省“转型项目建设年”活动的总体部署，强力抓好项目储备和实施，完善工作机制，拓宽融资渠道，营造良好环境，推动项目建设取得突破，为加快转型、推动高质量发展提供强劲动力。

　　主攻重大项目。落实国发42号和晋发49号文件精神，滚动储备一批大项目、好项目，做实“五十百千”项目盘子，百亿元项目达5个以上，十亿元项目达10个以上，亿元项目达100个以上，千万元项目达1000个以上。项目储备实行滚动管理、按季调度，及时补充优质项目入库，不断充实完善项目储备盘子。重点监测推进620个项目，总投资4178亿元，当年完成投资684亿元。强力推进154个省市重点项目，总投资2997亿元，当年完成投资328亿元。大力实施山西其亚240万吨氧化铝及铝镁电材一体化、山西焦煤60万吨烯烃、山西立恒40万吨乙二醇、华翔集团精密铸造及机加工智能化工厂、霍州兆光电厂固废物综合利用、蒲县肖家沟煤炭集运站项目等一批转型项目。

　　完善工作机制。健全完善领导包联、定期调度、双向协调等工作推进机制，最大限度减少核准事项和前置条件。强化要素配置，优先保障重大转型项目。切实加强项目调度，掌握项目进展，加强分析研判，发现和解决项目推进中的突出矛盾和问题。加强督查督办，推动项目建设取得实效。

　　拓宽融资渠道。充分发挥直接融资的主渠道作用，灵活运用银行贷款、发行债券等各类金融工具，多渠道解决项目融资难题。用好政府投资的产业基金，吸引创业投资、私募股权投资等各类资金参与项目建设。规范有序推进政府与社会资本合作，稳妥推进实施一批PPP项目，撬动社会资本，激发投资活力。

　　优化营商环境。扎实开展“六最”营商环境建设年活动，加快推动相对集中行政许可权试点，全面推进企业投资项目承诺制，推行清单告知、平台办理、流程再造、多图联审、收费管理“五统一”。继续做好干部入企服务工作，提供一对一、精准化的保姆式服务，做到有求必应，无需不扰。畅通政企沟通渠道，发挥行业协会商会作用，努力为企业投资、项目建设创造最优环境。

　　(四)深入推进生态治理

　　“绿水青山就是金山银山”。紧紧抓住我市纳入汾渭平原大气污染防治重点区域这一重大契机，围绕“天蓝、水清、地绿”，统筹山水林田湖草，坚持生态立市，打好四大战役，强化执法保障，推动生态环境质量持续改善。

　　打好蓝天保卫战。深度治理涉气企业，重点对电力、焦化、钢铁等企业无组织散放和白色烟羽进行治理，实现主要大气污染物特别限值排放。持续整治“散乱污”企业，分类采取关停、整合、升级、改造、转产、搬迁等措施，最大程度削减污染排放量。积极稳妥推进清洁取暖改造，综合采取工业企业余热利用、地热连片供暖、建设集中供热燃煤锅炉或生物质锅炉等方式，推进多元供热。落实《临汾市燃煤污染防治规定》，制定全市散煤管控方案，严控使用劣质散煤。强化机动车污染防治，基本实现市区和平川县(市、区)公交纯电动化、出租车燃料清洁化。深化餐饮油烟治理，开展市区露天摊点专项整治。强化扬尘治理，严格落实城市区域建筑工地6个100%措施，综合整治路域环境。

　　打赢水污染治理歼灭战。围绕“水质好起来、水量丰起来、风光美起来”的要求，推进水环境治理和生态改善。重点加强城市水源地保护工作，完成龙子祠水源地一级保护区和霍州市郭庄泉水源地环境综合整治工程。强化工业废水深度治理，焦化、医药、化工等企业排水稳定达到相应地表水质量标准。持续推进生活污水处理，加快实施老旧供水管网改造及排水管网雨污分流改造工程，全面实施城镇污水处理厂提标改造，达到地表水Ⅲ类标准。深入推行“河长制”，以汾河为重点，全面整治和规范入河排污口，加强排污源头监测和截污纳污管网建设，杜绝各类污水直排。推进流域生态治理，落实市管河流生态基流调度方案，保障和增加主要河流生态基流量，按期完成汾河公园、汾河尧都区吴村段、涝洰河、浍河、芝河、昕水河、州川河、鄂河等重点河流治理项目，昕水河水质稳定达地表水Ⅲ类标准，汾河水质达地表水Ⅴ类标准。强化地下水污染防治，完成全市所有加油站地下油罐防渗改造任务。

　　打响土壤污染防治持久战。制定全市土壤污染治理与修复规划，开展土壤污染调查与评估。提升土壤环境监测能力，完成土壤环境质量国控监测点位设置，逐步实现所有县(市、区)全覆盖。开展全市土壤和农产品协同调查，摸清农用地土壤污染基本情况，采取有力措施，开展防治工作。

　　打胜生态修复治理总体战。落实西山七县生态主体功能区定位，严格实行产业准入负面清单制度。大规模开展国土绿化行动，集中新一轮退耕还林、三北防护林、天然林资源保护、太行山绿化、黄土高原治理等国家和省级林业工程项目，加快建设吕梁山生态脆弱区、太行山水源涵养区、百里汾河经济带湿地植被恢复区三大生态屏障，完成营造林44.1万亩。推进实施东西两山淤地坝除险加固、固沟保塬、小流域综合治理等水土保持项目，新增水土流失治理面积65.7万亩。

　　强化环境监管执法保障。严格落实环境保护“党政同责、一岗双责、齐抓共管、失职追责”的要求，进一步强化环境保护县(市、区)属地责任和部门行业监管责任，健全完善部门联动、“一城三区”一体化治理、平川区域和流域上下游联防的工作机制。将环保工作列入年度目标责任考核重要内容，实行“一票否决”。从严落实省、市量化问责相关规定，对重点污染项目进行挂牌督办，严厉查处各类环境违法行为，对失职渎职、不作为乱作为的干部严肃追责问责。

　　各位代表，风雨多经志弥坚，关山初度路犹长。良好的生态环境是最公平的公共产品，是普惠的民生福祉。保护生态环境就是保护生产力，改善生态环境就是发展生产力。污染防治攻坚战只能打好打赢，没有退路。我们要像保护眼睛一样保护生态环境，像对待生命一样对待生态环境，坚定不移、持之以恒地打造生态环保“临汾品牌”，让绿水青山成为临汾永续发展的金山银山!

　　(五)扎实推进脱贫攻坚

　　坚持聚焦再聚焦，精准再精准，围绕三年行动计划，强化三个管理，落实五联五帮，推进十大工程，全力打好脱贫攻坚战。

　　强化三个管理。强化动态管理。扎实推进全市建档立卡数据清洗和贫困人口动态调整，线上线下保持一致。强化精细管理。实行脱贫攻坚工作周例会月报告季总结制度，全面压实市县乡村四级脱贫攻坚主体责任，严格落实包村领导、驻村工作队和第一书记“三支队伍”帮扶责任，因村因户因人施策。强化留痕管理。大力推行“五清单两标准一档案”工作，规范完善各类档案资料，夯实精准扶贫基础，提升脱贫质量成色。

　　落实五联五帮。聚焦深度贫困，健全完善帮扶机制，全面落实五联五帮责任制，每个深度贫困县由1名市级领导联系帮扶，1个经济强县结对帮扶，1个企业合作帮扶，1家市级医院、1所市级大中专或职业院校对口帮扶，不断完善具有临汾特色的大扶贫格局。

　　实施十大工程。特色产业扶贫工程。以干鲜果、畜牧、蔬菜、小杂粮等七大特色产业为主攻方向，加快培育、做大做强贫困地区主导产业。全面推进“一村一品一主体”，巩固提升“五有”成果。贫困村提升工程。瞄准制约贫困村的发展短板，继续大力实施农村危房改造、饮水安全、通村公路等基础设施建设，改善贫困村条件。易地扶贫搬迁工程。全面铺开105个深度贫困村整体搬迁和1.8万人易地扶贫搬迁工程，年底竣工60%以上。隰县、古县、蒲县、浮山等4个县的贫困户，全部搬迁入住。健康扶贫工程。全面落实“三保险三救助”帮扶政策，将住院医疗费用个人自付比例控制在10%以内。深化健康扶贫“双签约”服务，对大病和慢性病贫困患者实行分类救治。社会保障工程。扎实推进教育、民政、卫计、残联四方面24项帮扶政策进村入户，将符合条件的老年人、残疾人、重病患者、丧失劳动能力以及因学致贫、因病致贫等人口全部纳入社会保障。生态扶贫工程。实施“五个一批”林业生态建设扶贫工程，推广大宁购买式造林扶贫经验，实现生态改善与精准脱贫同步推进、同步见效。贫困县完成退耕还林14.1万亩、造林绿化35.1万亩、干果经济林提质增效20.3万亩，惠及7.1万人。光伏扶贫工程。重点实施70兆瓦集中式地面电站和73.2兆瓦的村级光伏扶贫电站，按时建成并网运行。加强村级电站运行管理和收益分配工作。金融扶贫工程。建立金融支持脱贫攻坚新机制，持续实施“富民贷”“强农贷”，抓住苹果期货上市并在我市吉县设立交割仓库的机遇，利用“保险+期货”“期货+订单”“保险+期权”等模式，推动产业扶贫、精准脱贫。扶志扶智工程。注重扶贫与扶志扶智结合，通过扶思想、扶观念、扶信心进行“扶志”，通过扶知识、扶技术、扶思路进行“扶智”，加快补齐贫困群众“精神短板”，激发脱贫内生动力。社会力量帮扶工程。坚持和完善“三位一体”大扶贫格局，引导社会力量助推脱贫攻坚，不断深化“百企帮百村”“百商帮百户”等活动，带动群众就业增收。

　　各位代表，精准扶贫，功在不舍;精准脱贫，志在必得。我们要加大财政投入，下足“绣花”功夫，确保隰县、蒲县、浮山、古县4个县“摘帽”、163个贫困村退出、4.9万贫困人口脱贫，尧都、霍州、襄汾、洪洞、翼城、曲沃等6个非贫困县(市、区)现有贫困人口整体脱贫，实现脱贫攻坚连战连胜!

　　(六)统筹推进乡村振兴

　　按照产业兴旺、生态宜居、乡风文明、治理有效、生活富裕的总要求，坚持规划先行，统筹推进五大振兴，加快农业农村现代化步伐。

　　坚持规划先行。编制市、县两级乡村振兴战略总体规划及产业兴旺、生态宜居、乡风文明、治理有效、生活富裕、体制机制创新六个专项规划，与“十三五”发展规划、土地利用规划、生态保护规划等有机衔接，以6个整体推进县、30个乡镇和100个村作为试点，通过示范引领，以点带面，推动工作。

　　推动产业振兴。围绕发展现代农业，推进产业融合，构建乡村产业体系。持续壮大粮食、蔬菜、水果、干果、中药材和畜牧等产业，粮食产量稳定在20亿公斤以上，杂粮种植面积稳定在95万亩以上，新增设施蔬菜1.4万亩，新发展和改造果园6万亩，新发展中药材6万亩，扩大肉鸡、晋南牛、生猪养殖规模，建设三大畜牧特色产业区。加快培育农产品加工龙头企业，打造2个国家级、7个省级和10个市级龙头企业，农产品加工销售收入达到100亿元以上。以城郊农业、休闲农业、乡村旅游为重点，加快田园综合体建设，促进三次产业深度融合。大力推进“三区两品牌”建设，打造国家级优质农产品出口示范区、黄土高原有机旱作农业先行区、临汾休闲农业全域旅游区“三大区域”，在继续推动吉县苹果走向全国、走向世界的基础上，着力打造叫响隰县玉露香梨和安泽连翘“两大品牌”。

　　推动人才振兴。围绕农民新型化、专业化、职业化的发展趋势，构建农村新型经营体系，培育专业大户、家庭农场、专业合作社等新型主体，提高对各类人才的吸引力。继续深入开展“引老乡、回故乡、建家乡”活动，建立健全激励机制，引导各类人才投身乡村振兴，培育有文化、懂技术、会经营的新型职业农民，打造一支强大的乡村振兴人才队伍。

　　推动文化振兴。加强农村精神文明建设，广泛开展文明村镇、星级文明户、文明家庭创建活动。以旅发大会举办为契机，传承弘扬优秀乡土文化，挖掘抢救优秀文化资源，加强传统古村落保护工作，积极发展民间艺术和传统手工技艺，打造农村品牌，提升文化附加值。加强村规民约建设，引导和组织农民成立红白理事会、村民议事会、道德评议会，推动移风易俗，树立文明新风。

　　推动生态振兴。深入推进卫生城镇创建、农村垃圾污水治理以及“厕所革命”，促进农村基础设施建设提档升级。持续推进美丽宜居乡村“三级联创”，通过以奖促治、政策引导、整合资源，推进省级15个美丽宜居乡村、2个集中连片区和市级25个美丽宜居乡村、3个集中连片区建设，完成美丽宜居乡村命名挂牌工作，打造农民安居乐业的美丽家园。

　　推动组织振兴。强化农村基层党组织领导核心地位，严格落实农村基层党建责任制，选优配强农村党组织书记。健全自治、法治、德治相结合的乡村治理体系，拓展“四议两公开”制度，落实“一村(社区)一法律顾问”制度，建立道德激励约束机制，让农村社会既充满活力又和谐稳定。

　　各位代表，山再高，往上攀，总能登顶;路再长，走下去，定能到达。乡村振兴战略是做好新时代“三农”工作的总抓手。我们要立足市情和农情，注重地域特色，体现乡土风情，强力推动农业全面升级、农村全面进步、农民全面发展，加快实现农业强、农村美、农民富!

　　(七)积极推进三产发展

　　三产兴则产业兴，三产强则转型成。坚持把文化旅游业打造成全市战略性支柱产业，加快发展生产性服务业，大力推进生活性服务业，不断提升贡献率，推动高质量发展。

　　加快发展文化旅游业。按照全域旅游理念，深入推进文化和旅游融合发展，办好一场盛会，实施两大工程，打造三大板块，加快基础设施建设，努力把我市打造成全国重要的旅游目的地。办好一场盛会，就是办好全省旅游发展大会。以“传统文化与新时代优质旅游融合创新”为主题，突出“3+N”活动，“3”是办好“华夏古文明·山西好风光”推介会、山西省旅游发展专家咨询委员会年会暨山西旅游创新才智峰会、山西文旅产业创新应用博览会3项省级会议;“N”是举办陶寺考古遗址发掘40周年国际论坛、大河文明国际旅游论坛、侯马盟书论坛等14项国家、省级重大活动及88项助力活动。实施两大工程，就是持续实施好平阳记忆、印象临汾两大文化工程。做好市博物馆展陈工作，唤醒城市记忆，彰显文化魅力。深入挖掘尧文化、晋文化、移民文化、商业文化、廉政文化、红色文化、山水文化等特色文化元素，推动文化与旅游融合发展。重点打造尧庙-华门文旅融合示范区。打造三大板块，就是全力打造黄河、根祖、太行三大旅游板块，推动旅游大开发。加快推进大槐树、壶口瀑布、云丘山国家5A级景区创建和人祖山、乾坤湾国家4A级景区创建，推动中镇霍山七里峪、曲沃晋国博物馆、尧都姑射山等一批景区提档升级。规划打造中线根祖文化游、东线太行山水游、西线黄河风情游等3条旅游精品线。加强基础建设，就是突出道路、厕所、停车场、旅游智慧平台、集散中心、景区水电气暖等基础设施建设，推动旅游基础设施提标升级。重点推进沿黄扶贫旅游公路项目建设，建成沿黄一号旅游公路永和乾坤湾试验段。加快推进尧都仙洞沟、襄汾陶寺、安泽红叶岭等7条旅游公路建设。启动实施永和、乡宁、吉县壶口等通用机场建设。

　　提速发展生产性服务业。发展现代物流。全力打造中西部现代商贸物流中心，充分发挥方略陆港集团多式联运示范工程项目带动辐射作用，加快推进临汾晋南国际农产品、侯马振通电子商务、传化智联、兴荣物流等园区建设。发展现代金融。加快多层次资本市场体系建设，大力发展股权投资、创业投资等基金。吸引更多的银行、保险等金融机构落户临汾，支持金融机构创新融资服务。加快推进县级农村信用社改制。提升信息技术服务。积极推广“互联网+”，开展信息惠民，创建新型智慧城市，加快推广应用“平阳一卡通”。

　　大力发展生活性服务业。发展家庭养老服务。积极培育一批管理规范、运作良好的家庭养老服务企业。积极引导支持民间资本参与，加快养老服务与相关产业融合发展，重点推进侯马怡之福老年医养中心项目建设。发展住宿餐饮服务。推进星级酒店提档升级，突出“温馨之旅”主题文化，加快推进金都花园、天鹅大酒店创建“五星级酒店”。推进“食品安全示范城市”创建，侯马、古县、大宁等县市完成创建任务。发展商贸流通业。围绕打造黄河金三角现代商贸中心，重点建设华夏国际商贸城、碧桂园城市综合体等项目。推进侯马、隰县、汾西“电子商务进农村综合示范县”建设。推广隰县电商模式，构建市县乡村四级联创机制。以侯马市为试点，推进跨境电子商务。

　　(八)大力推进基础建设

　　全面贯彻落实以人为核心的城镇化发展理念，统筹区域发展，坚持建管并重，加快交通水利、电网通讯等基础设施建设，推动城乡融合发展迈出新步伐。

　　完善区域规划。坚持统筹规划、优化布局，加快制定和完善百里汾河生态经济带、百里黄河绿色发展示范区、百里太岳可持续发展示范区“三个百里”互促共进、协调发展的大区域规划。全力加快《临汾市市域城镇体系规划(2017-2035)》《临汾市城市总体规划(2017-2035)》修编工作，完善全市城镇规划体系，充分发挥规划引领作用。扎实推进行政区划调整工作。

　　坚持建管并重。围绕“建设新城、改造老城、提升东城、绿色绕城”要求，着力提升河西新城城市规划设计，实施城市“双修”。推进实施道路、畅通、绿化、亮化、棚改、街巷改造等10大类119项城建项目。重点建设尧天大街等27条道路，打通尧贤街南延、体育街南延等10条“断头路”，建设水塔街、广宣街等10个游园和4处绿地，实施北城濠西段棚户区改造、河汾一路工程安置房等21项棚改工程，启动平阳大桥、马务锣鼓桥等亮化工程和市区路灯远程控制项目，不断提升承载能力和城市品位。加强城市精细化管理，建立数字化管理平台，全面整合执法队伍，整治违章建筑、马路市场、静态车辆乱停乱放等行为，创建干净整洁、规范有序的市容环境。

　　加快交通建设。以创建“全国性综合交通枢纽城市”为目标，全力加快高速公路建设，完成长临高速、霍永高速西段二期等工程，大力推进隰吉、洪大、古翼三条高速公路前期工作，力争年内开工建设;加快推进霍永高速东段蒲县连接线，长临高速古县、安泽、浮山、临汾机场连接线和东贾互通等工程。推进国道309线尧都区小榆至峪里等5条国省干线公路改造。扎实推进“四好农村路”建设，开展国家级、省级示范县创建工作。

　　加强水利建设。加速大水网骨干工程建设，重点实施引沁入汾浮山供水工程，力争年底完工。持续推进县域小水网配套建设，加快实施中部引黄蒲大支线延伸段工程，启动实施引沁入汾尧都专线、浮翼连接线等工程，续建尧都区仙洞沟、蒲县刁口等5座水库，争取新开工建设蒲县小水库、汾西县小水网等2项输水工程。提高跨河、跨区域调水能力。

　　升级电网和通讯网络。全面提升供电能力和通讯能力，重点推进临汾西500千伏输变电工程，启动洪洞南220千伏、古县220千伏、洪洞郭堡110千伏等输变电工程。加快通讯网络建设，超前谋划布局5G基站，推动互联网快速发展。

　　(九)切实推进总体安全

　　突出打好防范化解重大风险攻坚战，既防“黑天鹅”，也防“灰犀牛”，扎实推进政府债务和金融风险防控、安全生产、社会稳定等工作。

　　防范化解重大风险。严防政府债务风险。严控债务规模，制定限额分配方案，县级政府举债规模不得突破批准限额。制定出台《关于对债务风险化解的实施方案》《政府性债务风险应急处置预案》，防止盲目举债、过度举债，确保债务风险得到有效管控。将新增债券和置换债券收支全部纳入预算管理，防止债务规模随意扩大化。防控金融风险。落实稳健中性货币政策，保持货币信贷合理适度增长。加强互联网金融监管。防范和化解企业债务违约风险，严厉打击恶意逃废金融企业债务行为。降低银行不良贷款率，规范银行资产管理业务。严打非法集资行为。建立全市非法集资监测预警体系。开展集中宣传和排查，从源头遏制非法集资案件高发态势。依法稳妥处置各类非法集资案件，守住不发生区域性风险底线。

　　筑牢安全生产防线。明确各级党委政府领导责任、部门监管责任和企业主体责任，构建横向到边、纵向到底的安全生产责任体系。开展“安全生产执法年”活动，严格执行“四个一律”“五个一批”，严肃查处各类非法违法行为。强化安全生产专项整治，加强煤矿、非煤矿山、道路交通、危化品、地质灾害、森林防火、动物防疫、食品药品等重点行业领域安全风险管控和隐患排查治理。加大安全生产考核权重，严格落实“一票否决”，推动全市安全生产形势持续稳定好转。

　　全力维护社会稳定。健全完善社会治安防控体系，广泛开展“平安守护”群防群治活动。大力实施公安大数据战略，加强网络舆情监控，有效排查管控信访维稳、公共安全等领域不稳定因素，妥善处置各类突发事件。强力开展扫黑除恶专项斗争，严打黑恶势力犯罪，铲除“保护伞”，形成打击各类违法犯罪的高压态势。扎实推进“平安临汾”“法治临汾”建设，为全市转型发展创造安全和谐稳定的社会环境。

　　(十)稳步推进民生改善

　　人民对美好生活的向往就是我们的奋斗目标。我们要始终坚持人民立场，把群众拥护不拥护、赞成不赞成、高兴不高兴、答应不答应作为衡量一切工作得失的根本标准，着力解决好全市人民最关心最直接最现实的利益问题。

　　优先发展教育事业。坚持公益普惠发展学前教育，实施学前教育第三期行动计划，推进洪洞、霍州、乡宁学前教育改革发展省级试点工作，新改扩建公办幼儿园19所，认定普惠性民办幼儿园48所。推进全市县域内城乡义务教育一体化发展，尧都区和市直学校同步完成义务教育发展基本均衡认定任务，着力解决中小学课外负担重、“择校热”“大班额”等突出问题。做好新高考改革推进工作，支持高中学校多样化发展，推动民办普通高中标准化建设。完成大宁、永和、汾西、安泽、隰县等5个县职教中心建设任务，大力提高中职招生录取比例。全面提高我市特殊教育服务水平。支持和规范社会力量办学，进一步推进社区教育发展。深化教育体制机制改革，推进“校长职级制”和“教师县管校聘”试点。持续开展“三优”评选活动，全面加强师资队伍和师德师风建设。全力推进山西师大和师大临汾学院新校区建设。

　　加快发展卫生健康事业。大力推进健康临汾建设，深入开展提升医疗质量专项行动，力争年底前全市公立医院全部实施临床路径管理。健全“三医联动”长效机制，推动县级医疗集团持续健康运行。稳步提升中医药服务能力，创建中医药特色基层医疗卫生机构12家、中医馆10家。实施“百千万卫生人才培养工程”。推进国家卫生城镇创建工作，完成第一、二批创建县(市、区)申报工作。城乡医保财政补助标准每人提高40元，达到490元。

　　大力发展文化事业。积极培育和践行社会主义核心价值观，加强爱国主义教育，弘扬家庭美德、职业道德、社会公德。把握舆论导向，传播社会发展正能量，讲好临汾故事。实施文化惠民工程，开展广场文化消夏月等系列活动。加快文化基础设施建设，重点启动市广电中心和美术馆项目前期工作，加快推进平阳文化生态保护实验区建设，推动县级“四馆一场一院”工程，完成飞碟射击靶场建设。积极推进优秀传统文化、非物质文化遗产的传承发展，推动非遗、戏曲文化进校园进课堂。发展壮大广播电视、影视创作、演艺娱乐、动漫游戏等文化产业。做好市县方志年鉴全覆盖工作。广泛开展全民健身和竞技体育运动，举办“尧王杯”全国马拉松、全国五人制足球赛等“十大体育赛事”。

　　全力促进和保障就业。依托驻临教育培训机构和华翔集团等大型企业，实施“临汾技工”培训行动计划，打造知名技工品牌，确保“临汾技工”就业率达到80%以上。持续开展好就业援助专项行动，做好农村转移劳动力、城镇失业人员、残疾人等群体就业工作。扩大农民工就业，稳妥解决拖欠农民工工资问题。

　　保障群众住有所居。始终坚持房子是用来住的、不是用来炒的定位，解决好各类群体的住房问题。不断扩大公租房保障覆盖面，将新就业大学生和外来务工人员等“新市民”群体全部纳入保障范围。出台租赁补贴政策，年度发放城镇住房保障家庭租赁补贴5500户。加快政府投资公租房建设，年内分配率达到90%。制定出台人才住房保障办法，支持高校、开发区等利用自有资金、土地建设人才公寓。

　　完善社会保障体系。稳步提高社会保障待遇水平，推动机关事业单位养老保险制度改革入轨运行，提高退休人员基本养老金和城乡居民基本养老保险标准。健全失业保险待遇与物价水平联动调整机制，推进失能、半失能人员长期护理保险的落实。对经济困难的高龄与失能老年人、百岁以上老年人执行补贴新标准。调整城乡低保标准和优抚补助标准,足额安排困难群众各类救助资金，加强对特困人员、残疾人等群体的兜底保障。

　　在做好上述工作的同时，还要深入开展双拥共建活动，做好国防动员、人民防空、民兵预备役工作;支持工会、妇联、共青团、工商联等人民团体工作;统筹审计、统计、档案、气象、水文、地震、地方志、民族宗教、外事侨务、对台、老龄、残疾人和关心下一代等各项工作;继续做好对口援疆工作。

　　各位代表，民有所呼，必有所应。我们要顺应人民期待，多办民生实事，增进民生福祉，让人民群众在共建共享中有更多获得感、幸福感和安全感。今年，市政府将在完成好省政府“六件民生实事”任务的基础上，办好10件民生实事：

　　一是落实《临汾市2018-2020年市区学校建设三年行动计划》，新、改、迁建中小学校10所。

　　二是对边远和贫困地区乡村医生补助标准每人每月增加200元。

　　三是为全市农村放映公益电影3.5万场，为农村寄宿制学校放映爱国主义教育影片3800场，免费送戏下乡1000场。

　　四是完成鼓楼东大街、广宣街、平阳街等30条市区主次干道人行道维修改造。

　　五是出台我市停车位管理办法，建设立体停车设施10座。

　　六是实施农村饮水安全巩固提升工程，保障农村群众吃上安全水、放心水。

　　七是实施0-6岁残疾儿童康复训练救助，对残疾人开展职业技能培训并帮扶就业。

　　八是新建改建“四好农村路”2000公里。

　　九是新开工棚户区(城中村)改造46273套(户)，基本建成6300套(户)，完成1192户农村地质灾害治理搬迁任务。

　　十是新建农村老年人日间照料中心50个。

　　三、建设人民满意的服务型政府

　　时代是出卷人，我们是答卷人，人民是阅卷人。我们要始终坚持与人民心心相印，与人民同甘共苦，与人民团结奋斗，努力建设人民满意的服务型政府。

　　坚持强化政治引领。牢固树立“四个意识”，始终坚定“四个自信”，坚决做到“四个服从”，坚决落实“三个一以贯之”，坚决维护以习近平同志为核心的党中央权威和集中统一领导，始终在政治立场、政治方向、政治原则、政治道路上，同以习近平同志为核心的党中央保持高度一致，做到思想上高度认同，政治上坚决拥护，组织上自觉服从，行动上紧紧跟随。落实意识形态工作责任制，加强阵地建设和管理。深刻学习领会习近平新时代中国特色社会主义思想，提高政治觉悟，增强政治定力，不折不扣贯彻中央、省委和市委各项决策部署。

　　全面推进依法行政。坚持法治思维，加强对新修订《宪法》的学习，严格依宪执政、依法行政，加快建设法治政府，把政府活动全面纳入法治化轨道。坚持科学决策、民主决策、依法决策，加快构建决策科学、执行坚决、监督有力的权力运行机制。依法接受人大及其常委会的法律监督、工作监督，自觉接受人民政协的民主监督，认真听取人大代表、政协委员意见，认真办理人大代表建议、政协委员提案，加强与民主党派、工商联、无党派人士和各人民团体的联系沟通。加强行政复议与应诉工作，健全政务公开工作考核评价机制，加强医疗、卫生、教育、社保等公共服务领域的信息公开，自觉接受人民群众的监督。

　　切实提高行政效能。全面深化、拓展、延伸“13710”工作制度，健全首问负责、一次性告知等制度，完善“清零”机制和办法，强化督查结果运用。加快推广OA办公系统，年内实现市县乡三级覆盖。继续压减和下放行政许可事项，编制公共服务事项清单，大力推行政务服务一窗受理、一网办理、一个机构管理、一枚印章审批，让数据“多跑路”，群众和企业“少跑腿”。加强政府诚信建设，规范政府采购、招投标、招商引资等行为。全面推进“双随机、一公开”，完善守信联合激励和失信联合惩戒机制，营造诚信政务环境。稳步推进政府系统机构改革，坚持做到队伍不乱、人心不散、工作不断。

　　强化能力作风建设。政府工作人员既要有担当的宽肩膀、硬肩膀，更要有成事的真本领、强本领。切实加强政府工作人员的专业素养和专业能力建设，持续开展领导干部能力提升培训，着力增强学习本领、政治领导本领、改革创新本领、科学发展本领、依法执政本领、群众工作本领、狠抓落实本领、驾驭风险本领。健全完善正向激励、考核评价、容错纠错机制，旗帜鲜明地为敢于担当、踏实做事、不谋私利的干部撑腰鼓劲。持续加强作风建设，狠刹“四风”，力戒形式主义、官僚主义，严厉整治干部不作为、慢作为、乱作为和选择性作为。继续深入开展领题调研活动，大兴调查研究之风，做到问题在一线解决，思路在一线形成，工作在一线推动。

　　从严抓好政治生态。落实全面从严治党的要求，始终坚持严字当头，一严到底，拧紧责任链条，层层传导压力，推动政府系统全面从严治党向纵深发展。扎实开展“不忘初心、牢记使命”主题教育，深入推进“两学一做”学习教育常态化制度化，巩固提升“维护核心见诸行动”主题教育成果，认真组织开展“强化责任、严守纪律、树好形象”专项治理活动。坚持把纪律和规矩挺在前面，严肃党内政治生活，使党员干部心中有戒，行有所止，守住底线，不碰高压线。坚决惩治各类腐败行为，紧盯重点领域和关键环节，加大案件查办力度，重点查处群众身边的腐败问题，真正把权力关进制度的笼子，努力为转型发展营造良好的政治生态。

　　各位代表，信仰就是灵魂，使命高于生命，责任重于泰山。新时代是奋斗者的时代。让我们高举习近平新时代中国特色社会主义思想的伟大旗帜，在市委的坚强领导下，在市人大、市政协和社会各界的监督支持下，把全市448万人民对美好生活的向往作为我们的奋斗目标，为人民不懈奋斗、同人民一起奋斗，凝心聚力，攻坚克难，奋力开创全市转型发展新局面，为决胜全面小康、实现振兴崛起，建设富裕文明绿色幸福美丽新临汾做出新的更大的贡献!
